# Supplementary material for: The Equine Dental Pulp: Analysis of the Stratigraphic Arrangement of the Equine Dental Pulp in Incisors and Cheek Teeth
Source: Vet Sci. 2022 Oct 30;9(11):602. doi: 10.3390/vetsci9110602 (PMC9695782; doi:10.3390/vetsci9110602)
Supplement: Supplementary file 1 [file vetsci-09-00602-s001.zip › vetsci-1980795-supplementary.pdf]

Table S1. Additional statistical significances of blood vessels with a diameter < 10 µm per 2000 µm² and measuring 10-50 µm per 2000 µm² in incisors and cheek teeth in different age groups (AG), regarding the defined zones (1.a to 4).

blood vessels	*age group*	*incisors*	*cheek teeth*
<10 µm	AG 1	Zone 1 vs. Zone 2: $p = 0.043$<br>Zone 1 vs. Zone 3: $p = 0.034$<br>Zone 1 vs. Zone 4: $p = 0.016$	not significant
	AG 2	not significant	not significant
	AG 3	not significant	not significant
10-50 µm	AG 1	Zone 1.a vs. Zone 2: $p = 0.008$<br>Zone 1.b vs. Zone 2: $p = 0.008$	Zone 1.a vs. Zone 3: $p = 0.021$<br>Zone 1.b vs. Zone 3: $p = 0.021$<br>Zone 1 vs. Zone 3: $p = 0.027$
	AG 2	Zone 1.a vs. Zone 2: $p < 0.001$<br>Zone 1.a vs. Zone 3: $p = 0.001$<br>Zone 1.b vs. Zone 2: $p < 0.001$<br>Zone 1.b vs. Zone 3: $p = 0.001$<br>Zone 1 vs. Zone 2: $p = 0.002$<br>Zone 1 vs. Zone 3: $p = 0.018$<br>Zone 2 vs. Zone 4: $p = 0.031$	Zone 1.a vs. Zone 3: $p = 0.032$<br>Zone 1 a vs. Zone 4: $p = 0.029$
	AG 3	not significant	not significant

Table S1.1 Additional statistical significances of blood vessels < 10 µm per 2000 µm² in the defined zones (1.a to 4) of incisors and cheek teeth, regarding different age groups (AG).

zones	*incisors*	*cheek teeth*
1.a	not significant	not significant
1.b	not significant	not significant
1	not significant	not significant
2	not significant	AG 1 vs. AG 3: $p = 0.002$<br>AG 2 vs. AG 3: $p = 0.02$
3	not significant	not significant
4	not significant	not significant

Table S2. Additional statistical significances of nerve fibers per 2000µm^2 in incisors and cheek teeth, regarding different age groups (AG) within the defined zones (1.a to 4).

zones	*incisors*	*cheek teeth*
1.a	AG 1 vs. AG 3: $p = 0.003$	not significant
1.b	not significant	not significant
1	not significant	AG 1 vs. AG 3: $p = 0.033$
2	not significant	AG 1 vs. AG 3: $p = 0.005$<br>AG 2 vs. AG 3: $p = 0.012$
3	not significant	AG 1 vs. AG 3: $p < 0.001$<br>AG 2 vs. AG 3: $p < 0.001$
4	not significant	not significant

Table S2.1 Additional statistical significances of nerve fibers per 2000µm^2 in incisors and cheek teeth, regarding different horizontal levels *so* (subocclusal), *c* (central) and *a* (apical) within the age groups (AG 1 to 3).

age group	*zone*	*incisors*	*cheek teeth*
AG 1	-	not significant	not significant
AG 2	global	not significant	so vs. a: $p = 0.03$
AG 3	within 3	so vs. c: $p = 0.002$<br>so vs. a: $p = 0.002$	so vs. c: $p < 0.001$<br>so vs. a: $p < 0.001$

Table S2.2 Additional statistical significances of nerve fibers per 2000µm^2 in incisors and cheek teeth, regarding defined zones (1.a to 4) within the age groups (AG 1 to 3).

age group	*incisors*	*cheek teeth*
AG 1	not significant	not significant
AG 2	not significant	Zone 1.a vs. Zone 4: $p = 0.03$
AG 3	not significant	not significant

Table S3. Additional statistical significances of fibroblastic cell nuclei per 2000µm^2 in incisors and cheek teeth regarding different age groups (AG) within the defined zones (1.a to 4).

zones	*incisors*	*cheek teeth*
1.a	not significant	AG 1 vs. AG 3: p = 0.026
1.b	not significant	AG 1 vs. AG 2: p = 0.023<br>AG 1 vs. AG 3: p = 0.015
1	not significant	AG 1 vs. AG 3: p = 0.037
2	not significant	not significant
3	not significant	AG 1 vs. AG 3: p = 0.026
4	not significant	AG 1 vs. AG 3: p = 0.018

Table S3.1 Additional statistical significances of fibroblastic cell nuclei per 2000µm^2 in incisors and cheek teeth, regarding different horizontal levels *so* (subocclusal), *c* (central) and *a* (apical) within the age groups (AG 1 to 3).

age group	*zone*	*incisors*	*cheek teeth*
AG 1	within 1	not significant	so vs. a: p = 0.014
AG 2	global	so vs. c: p = 0.028<br>so vs. a: p = 0.008	not significant
AG 3	within 2	so vs. c: p = 0.002<br>so vs. a: p = 0.002	not significant

Table S3.2 Additional statistical significances of fibroblastic cell nuclei per 2000µm^2 in incisors and cheek teeth, regarding the defined zones (1.a to 4) within the age groups (AG 1 to 3).

age group	*incisors*	*cheek teeth*
AG 1	not significant	within c:<br>Zone 1.b vs. Zone 2: p = 0.012<br>Zone 1.b vs. Zone 3: p = 0.023<br>Zone 1.b vs. Zone 4: p = 0.021
AG 2	Zone 1.a vs. Zone 2: p = 0.002<br>Zone 1.a vs. Zone 3: p = 0.002<br>Zone 1.a vs. Zone 4: p = 0.002<br>Zone 1.b vs. Zone 2: p = 0.003<br>Zone 1.b vs. Zone 3: p = 0.002<br>Zone 1.b vs. Zone 4: p = 0.003	Zone 1.a vs. Zone 1.b: p = 0.026<br>Zone 1.a vs. Zone 2: p = 0.002<br>Zone 1.a vs. Zone 3: p = 0.002<br>Zone 1.a vs. Zone 4: p = 0.002

	Zone 1 vs. Zone 2: $p = 0.048$<br>Zone 1 vs. Zone 3: $p = 0.029$<br>Zone 1 vs. Zone 4: $p = 0.043$	
AG 3	Zone 1.b vs. Zone 3: $p = 0.031$<br>Zone 1.b vs. Zone 4: $p = 0.043$	not significant

Table S3.3 Additional detailed statistical significances of fibroblastic cell nuclei per 2000μm^2 in incisors and cheek teeth, regarding the defined zones (1.a to 4) within a specific horizontal level (*so*, *c*, *a*).

age group	incisors	cheek teeth
AG 1	not significant	within central (*c*):<br>Zone 1.a vs. Zone 2: $p = 0.012$<br>Zone 1.a vs. Zone 3: $p = 0.023$<br>Zone 1.a vs. Zone 4: $p = 0.02$ 1
AG 2	not significant	within subocclusal (*so*):<br>Zone 1.a vs. Zone 1.b: $p = 0.009$<br>Zone 1.a vs. Zone 2: $p = 0.001$<br>Zone 1.a vs. Zone 3: $p < 0.001$<br>Zone 1.a vs. Zone 4: $p < 0.001$<br>wihtin apical (*a*):<br>Zone 1.a vs. Zone 1.b: $p = 0.035$<br>Zone 1.a vs. Zone 1: $p = 0.046$<br>Zone 1.a vs. Zone 2: $p = 0.002$<br>Zone 1.a vs. Zone 3: $p = 0.005$<br>Zone 1.a vs. Zone 4: $p = 0.027$
AG 3	within subocclucal (*so*):<br>Zone 1.a vs. Zone 3: $p = 0.031$<br>Zone 1.a vs. Zone 4: $p = 0.032$<br>Zone 1.b vs. Zone 3: $p = 0.006$<br>Zone 1.b vs. Zone 4: $p = 0.006$<br>Zone 1 vs. Zone 3: $p = 0.047$<br>Zone 1 vs. Zone 4: $p = 0.048$	not significant
